# Real-World Prescribing Patterns and Factors Associated with In-Hospital Statin Initiation Among Statin-Naïve Patients with Acute Decompensated Heart Failure: The HEROES Registry

**DOI:** 10.3390/jcm15155908

**Published:** 2026-07-29

**Authors:** Mateusz Lucki, Bogna Grygiel-Górniak, Ewa Lucka, Przemysław Mitkowski, Ewa Straburzyńska-Migaj, Marek Grygier, Robert Morawiec, Agnieszka Kapłon-Cieślicka, Agata Galas, Katarzyna Byczkowska, Piotr Hamala, Maciej Lesiak

**Affiliations:** 1 Department of Cardiology, Poznan University of Medical Sciences, 61-701 Poznan, Poland; mateusz.lucki@ump.edu.pl (M.L.); przemyslaw.mitkowski@ump.edu.pl (P.M.); estraburzynskamigaj@ump.edu.pl (E.S.-M.); marek.grygier@ump.edu.pl (M.G.); maciej.lesiak@ump.edu.pl (M.L.); 2 Department of Rheumatology, Rehabilitation and Internal Diseases, Poznan University of Medical Sciences, 61-701 Poznan, Poland; bgrygiel@ump.edu.pl; 3 Clinical Rehabilitation Laboratory, Department of Rehabilitation and Physiotherapy, Poznan University of Medical Sciences, 61-701 Poznan, Poland; 4 2nd Department of Cardiology, Medical University of Lodz, 90-419 Lodz, Poland; robert.morawiec@umed.lodz.pl; 5 1st Department of Cardiology, Medical University of Warsaw, 02-091 Warszawa, Poland; agnieszka.kaplon@gmail.com; 6 Department of Cardiology and Internal Diseases, Military Institute of Medicine, 01-755 Warsaw, Poland; agalas@wim.mil.pl; 7 National Institute of Cardiology, 04-628 Warsaw, Poland; kbyczkowska@ikard.pl; 8 1st Department of Cardiology, Medical University of Lodz, 90-419 Poznan, Poland; piotrhamala@gmail.com

**Keywords:** acute decompensated heart failure, LDL cholesterol, lipid-lowering therapy, prescribing patterns, statins

## Abstract

**Background:** This study aimed to characterize real-world patterns of in-hospital statin initiation and identify clinical characteristics associated with this prescribing decision among previously statin-naïve patients hospitalized for acute decompensated heart failure (ADHF) in the Polish HEROES registry. **Methods:** We analyzed 374 statin-naïve patients hospitalized for ADHF, including 168 patients in whom statin therapy was initiated during the index hospitalization and 206 in whom it was not initiated. Demographic, clinical, laboratory, echocardiographic, functional and treatment-related variables were compared between the groups. A prespecified forced-entry multivariable logistic regression model including 10 clinically relevant and nonredundant covariates was used to evaluate factors associated with statin initiation. **Results:** After multivariable adjustment, higher odds of statin initiation were associated with male sex (adjusted odds ratio [aOR], 2.01; 95% confidence interval [CI], 1.12–3.61), arterial hypertension (aOR, 1.65; 95% CI, 1.06–2.56), previous myocardial infarction (aOR, 1.82; 95% CI, 1.03–3.22), higher systolic blood pressure (aOR per 10 mmHg increase, 1.12; 95% CI, 1.01–1.25), higher LDL-C concentration (aOR per 10 mg/dL increase, 1.15; 95% CI, 1.07–1.22), and anticoagulant therapy (aOR, 1.88; 95% CI, 1.20–2.96). Increasing age (aOR per 1-year increase, 0.97; 95% CI, 0.95–0.99) and a higher KCCQ-12 score (aOR per 10-point increase, 0.88; 95% CI, 0.81–0.96) were associated with lower odds of treatment initiation. LVEF and the GDMT score were not significantly associated with statin initiation. **Conclusions:** In previously statin-naïve patients hospitalized for ADHF, statin initiation reflected a range of clinical and treatment-related characteristics. These variables should be interpreted as correlates of real-world physician-guided prescribing decisions rather than as causal determinants of statin initiation or predictors of clinical benefit. The study does not provide evidence supporting routine statin initiation solely because of ADHF.

## 1. Introduction

Heart failure (HF) remains a major global public health burden, affecting more than 64 million individuals worldwide and accounting for substantial morbidity, mortality, and healthcare expenditure [1]. Despite advances in pharmacological and device-based treatment, patients hospitalized for acute decompensated heart failure (ADHF) remain at high risk of readmission and death following discharge [2]. Hospitalization therefore provides an important opportunity not only to optimize HF-specific therapy but also to reassess concomitant cardiovascular disease, modifiable risk factors, and long-term preventive treatment. Unlike routine outpatient encounters, the inpatient setting enables a structured review of the patient’s medication history, atherosclerotic cardiovascular disease status, lipid profile, renal function, diabetes, and other determinants of cardiovascular risk. It also creates a clearly defined transition-of-care point at which preventive therapy can be reconsidered and, when appropriate, initiated, documented, and incorporated into the discharge plan [3].

Statins—3-hydroxy-3-methylglutaryl coenzyme A reductase inhibitors—are a cornerstone of cardiovascular prevention because of their LDL-C-lowering effects and proven benefits in patients with established atherosclerotic cardiovascular disease (ASCVD) [4] or sufficiently elevated cardiovascular risk [5]. Recent observational evidence has also evaluated the initiation of statins for primary prevention in patients with heart failure with preserved ejection fraction (HFpEF) [6]. However, two landmark randomized controlled trials, CORONA and GISSI-HF, did not demonstrate significant reductions in mortality or major cardiovascular outcomes when statins were evaluated specifically as treatment for chronic systolic HF [7,8]. Consequently, current European and American guidelines do not recommend routine initiation of statin therapy solely because a patient has HF [3,9]. In patients with HF, the decision to initiate a statin should instead be based on concomitant ASCVD, dyslipidemia, diabetes mellitus, chronic kidney disease, and overall cardiovascular risk.

Although observational studies have evaluated associations between statin use and clinical outcomes in patients with heart failure [10,11,12], limited evidence is available on how preventive recommendations are translated into real-world in-hospital prescribing decisions among previously statin-naïve patients with ADHF. This population is particularly relevant for an implementation analysis because initiation of treatment can be directly observed without the confounding influence of continuation of pre-existing statin therapy. Moreover, during an acute HF hospitalization, immediate hemodynamic stabilization and optimization of HF-specific treatment may take precedence over the reassessment of long-term cardiovascular prevention.

Several characteristics assessed in the present analysis, including LDL-C concentration, previous myocardial infarction, and cardiovascular risk category, are established components of guideline-based statin decision-making [3,9,13]. Accordingly, the purpose of this study was not to identify novel biological or mechanistic predictors of statin use, but rather to determine how registry-identifiable preventive indications and other patient characteristics were reflected in actual in-hospital prescribing decisions. The analysis therefore addresses treatment patterns and the implementation of preventive recommendations rather than the clinical effectiveness of statins in ADHF.

The primary objective was to determine the frequency of statin initiation among previously statin-naïve patients hospitalized for ADHF and to identify demographic, clinical, biochemical, and treatment-related characteristics independently associated with this decision. The secondary objective was to assess the alignment between in-hospital statin initiation and registry-identifiable secondary- or primary-prevention indications. Additional analyses compared cardiovascular risk profiles and the use of concomitant guideline-directed HF therapies between patients in whom statin therapy was and was not initiated during the index hospitalization.

## 2. Materials and Methods

### 2.1. Study Design and Population

This study was a retrospective observational analysis of prospectively collected data from the HEROES study (HEart failuRe ObsErvational Study), a multicenter registry initiated by the Polish Cardiac Society [14]. The registry enrolled adult patients with HF between 2 April 2022 and 27 March 2024. Participating centers included tertiary referral and academic hospitals experienced in the management of patients with HF.

Medical records of 1442 adult patients with heart failure were initially screened. After the exclusion of 349 patients managed exclusively as outpatients, 1093 hospitalized patients remained eligible for further evaluation. Of these, 74 patients were excluded because of an incomplete lipid profile, leaving 1019 patients with the data required for the present analysis. A further 645 patients receiving statin therapy before hospital admission were excluded.

**Figure 1:**
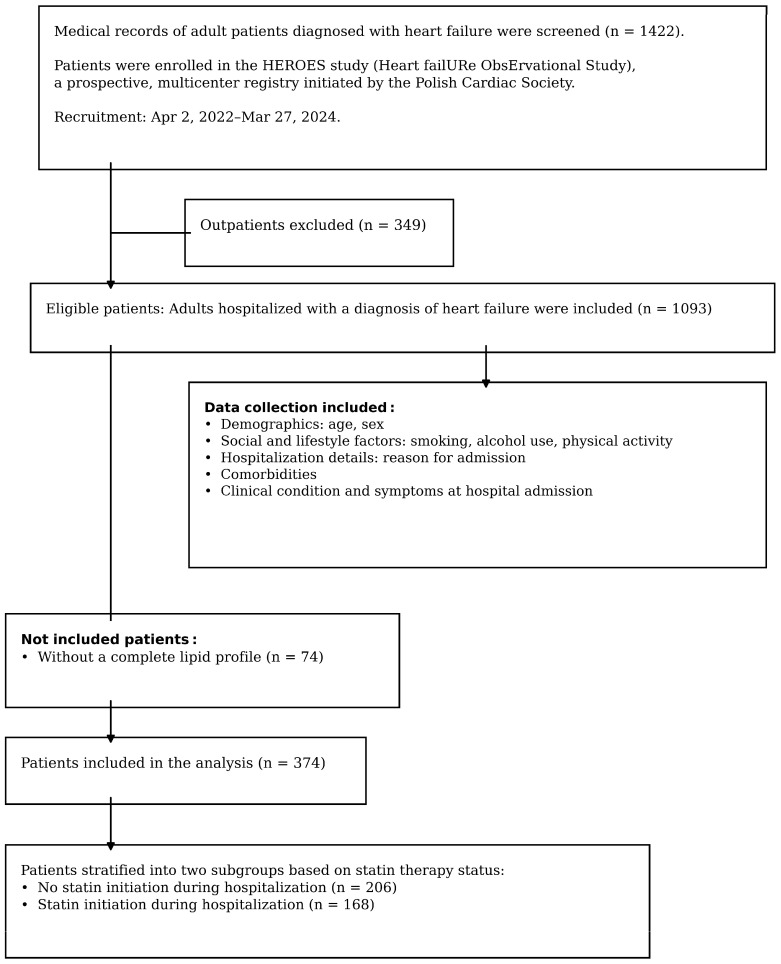
Flowchart of patient selection and grouping according to in-hospital statin initiation.

The final study population therefore comprised 374 patients who were statin-naïve at admission. In-hospital statin initiation was defined as a newly documented statin prescription during the index hospitalization in a patient who had not been receiving a statin before admission. Statin therapy was not initiated in 206 patients, whereas it was initiated in 168 patients. The patient-selection process and study-group allocation are presented in Figure 1.

The primary study outcome was in-hospital statin initiation. The analysis was designed to characterize real-world treatment patterns and patient characteristics associated with this prescribing decision. It was not designed to estimate the causal effect or clinical effectiveness of statin therapy in patients hospitalized for acute decompensated heart failure.

### 2.2. Variables Included in the Analysis

The analysis included demographic, lifestyle, clinical, biochemical, echocardiographic, functional, and treatment-related variables recorded during the index hospitalization.

Demographic and lifestyle variables included age, sex, body mass index, alcohol consumption, and smoking status, categorized as current, former, or never smoking. Clinical measurements included systolic and diastolic blood pressure, heart rate, and change in body weight during hospitalization. Functional status was assessed using the New York Heart Association functional classification, Canadian Cardiovascular Society angina classification, and Timed Up and Go test.

The evaluated comorbidities included arterial hypertension; atrial fibrillation, categorized as paroxysmal or persistent/permanent; ischemic heart disease; previous myocardial infarction; stable angina; previous percutaneous coronary intervention; previous coronary artery bypass grafting; carotid artery disease; lower-extremity artery disease; limb amputation; diabetes mellitus; chronic kidney disease, including dialysis dependence; anemia; iron deficiency; stroke; transient ischemic attack; thyroid dysfunction; depression; and malignancy.

Laboratory parameters included lipid profile variables (total cholesterol, LDL-C, HDL-C, and triglycerides), glucose metabolism markers (fasting glucose and HbA1c), renal function (creatinine, sodium, potassium, and uric acid), inflammatory and infection markers (CRP, WBC, procalcitonin), liver enzymes (ALT, AST, bilirubin), hematologic indices (hemoglobin, platelets), and cardiac biomarkers (NT-proBNP and troponin I).

Heart failure etiology was categorized as ischemic heart disease, cardiomyopathy, hypertensive heart disease, arrhythmia-induced cardiomyopathy, suspected toxic cardiomyopathy, significant valvular heart disease, or other causes. Echocardiographic assessment included left ventricular ejection fraction (LVEF). Patients were classified as having heart failure with preserved ejection fraction (HFpEF; LVEF ≥ 50%), heart failure with mildly reduced ejection fraction (HFmrEF; LVEF 41–49%), or heart failure with reduced ejection fraction (HFrEF; LVEF ≤ 40%).

Patient-reported health status was assessed using the Kansas City Cardiomyopathy Questionnaire-12 summary score and a visual analogue quality-of-life scale.

Pharmacological treatment recorded before or at hospital admission included angiotensin-converting enzyme inhibitors (ACEIs), angiotensin receptor blockers (ARBs), angiotensin receptor–neprilysin inhibitors (ARNIs), mineralocorticoid receptor antagonists (MRAs), β-blockers, diuretics, sodium–glucose cotransporter 2 inhibitors, antiplatelet agents, anticoagulants, and nitrates. Treatment administered during hospitalization included diuretics, inotropic and vasoactive agents, and respiratory or mechanical circulatory support.

### 2.3. Guideline-Directed Medical Therapy Score

To provide a parsimonious summary of baseline heart-failure-directed pharmacotherapy and to avoid entering several potentially correlated medication variables separately into the regression model, a guideline-directed medical therapy score was constructed. One point was assigned for the documented use of each of the following four treatment components before or at hospital admission: (1) renin–angiotensin system inhibition with an angiotensin-converting enzyme inhibitor, angiotensin receptor blocker, or angiotensin receptor–neprilysin inhibitor; (2) a beta-blocker; (3) a mineralocorticoid receptor antagonist; (4) a sodium–glucose cotransporter 2 inhibitor.

Angiotensin-converting enzyme inhibitors, angiotensin receptor blockers, and angiotensin receptor–neprilysin inhibitors were treated as a single mutually exclusive therapeutic component. The resulting score ranged from 0 to 4 points, with higher values indicating a greater number of guideline-directed treatment components.

Anticoagulant therapy was not included in the GDMT score and was analyzed as a separate variable. The score was intended to characterize the number of documented treatment components and should not be interpreted as a formal measure of guideline adherence, because information on individual drug eligibility, contraindications, tolerance, achieved dose, and reasons for non-prescription was not systematically available.

### 2.4. Cardiovascular Risk Classification

Cardiovascular risk was reassessed using a hierarchical and mutually exclusive approach. Patients with documented atherosclerotic cardiovascular disease were classified as being at very high cardiovascular risk. Among patients without established atherosclerotic cardiovascular disease, cardiovascular risk was classified on the basis of diabetes mellitus, chronic kidney disease, other relevant clinical conditions, and cardiovascular risk information available in the registry. Each patient was assigned to one category only: very high, high, or low/moderate cardiovascular risk.

An additional hierarchical and mutually exclusive patient-level classification was used to characterize registry-identifiable indications for lipid-lowering treatment. Patients with documented atherosclerotic cardiovascular disease were classified as having a secondary-prevention indication irrespective of baseline LDL-C concentration. Established atherosclerotic cardiovascular disease included documented coronary artery disease, previous myocardial infarction, previous percutaneous coronary intervention or coronary artery bypass grafting, ischemic stroke, lower-extremity artery disease, or documented carotid atherosclerotic disease.

Among patients without established atherosclerotic cardiovascular disease, a registry-identifiable primary-prevention indication was assessed using cardiovascular risk category, baseline LDL-C concentration, diabetes mellitus, chronic kidney disease, and other prespecified information available in the registry. Patients who did not meet the prespecified criteria for either a secondary- or primary-prevention indication were classified as having no preventive indication identifiable from the available registry data. Each patient was assigned to one category only.

These retrospective classifications were used to characterize the clinical context in which treatment decisions were made. They were not intended to replace a comprehensive prospective cardiovascular risk assessment or to adjudicate the appropriateness of individual prescribing decisions.

Cardiovascular risk category and registry-identifiable indication category were not entered into the primary multivariable regression model. Both were composite variables constructed partly from clinical characteristics already represented in the model, including previous myocardial infarction, atherosclerotic cardiovascular disease, LDL-C concentration, diabetes mellitus, and chronic kidney disease. Their simultaneous inclusion with component variables could have introduced conceptual circularity, redundancy, and unstable interpretation of the mutually adjusted coefficients.

### 2.5. Statistical Analysis

Statistical analysis was performed using PQStat software (version 1.8.6.126; PQStat Software, Poznań, Poland). The distribution of continuous variables was assessed using the Shapiro–Wilk test and inspection of their distributions. Normally distributed continuous variables were presented as mean ± standard deviation, whereas non-normally distributed variables were expressed as median and interquartile range. Categorical variables were summarized as absolute counts and percentages.

Comparisons between patients in whom statin therapy was and was not initiated were performed using Student’s *t*-test for normally distributed continuous variables, the Mann–Whitney U test for non-normally distributed continuous variables, and the chi-square test or Fisher’s exact test for categorical variables, as appropriate.

A multivariable binary logistic regression model was used to evaluate patient characteristics associated with in-hospital statin initiation. Statin initiation was coded as 1 and non-initiation as 0. The primary analysis used a fixed Enter model in which all the selected covariates were entered simultaneously. All the covariates included in the multivariable model were selected a priori based on clinical relevance and their representation of distinct, nonredundant clinical domains, rather than solely according to statistical significance in univariable comparisons. No automated stepwise selection procedure was used for the primary model.

The prespecified model included 10 covariates: age; male sex; arterial hypertension; history of myocardial infarction; systolic blood pressure; LDL-C concentration; left ventricular ejection fraction; KCCQ-12 summary score; GDMT score; anticoagulant therapy.

LDL-C was selected as the principal lipid variable. Total cholesterol was not entered simultaneously because of its biological and statistical overlap with LDL-C. Similarly, the individual components of the GDMT score were not entered together with the composite score. Cardiovascular risk category was not included because it was partly derived from clinical and laboratory variables already represented in the model.

Continuous variables were scaled to clinically interpretable units. Adjusted odds ratios were reported per 1-year increase in age; per 10 mmHg increase in systolic blood pressure; per 10 mg/dL increase in LDL-C; per 5-percentage-point increase in LVEF; per 10-point increase in KCCQ-12 score; and per one-component increase in the GDMT score. Binary variables were coded as present versus absent.

The regression analysis was based on complete cases. Missing observations were not imputed and were not coded as absence of a condition or treatment. Patients with a missing value for any variable included in the final model were excluded from the multivariable analysis. The complete-case sample included 374 patients, of whom 168 underwent in-hospital statin initiation.

The final model contained 10 covariates, excluding the intercept. The events-per-variable ratio was calculated by dividing the number of statin-initiation events in the complete-case sample by 10 and was 16.8. All the variables included in the model are reported in irrespective of statistical significance.

The overall significance of the model was assessed using a likelihood-ratio test comparing the full model with the intercept-only model. The full model provided a significantly better fit than the intercept-only model (likelihood-ratio χ^2^(10) = 29.59, *p* < 0.001).

Linearity in the logit was assessed for continuous predictors using the Box–Tidwell procedure. Interaction terms between each continuous predictor and its natural logarithm were added to the model. A statistically significant interaction term (*p* < 0.05) was considered evidence of departure from linearity in the logit. The Box–Tidwell procedure was applied only to variables with strictly positive values. Because the GDMT score was an ordinal variable ranging from 0 to 4, it was evaluated separately by comparing a model in which the score was entered as a single linear term with a model in which it was entered as a categorical variable. No statistically significant departures from linearity in the logit were identified for the continuous predictors included in the model.

The results are presented as adjusted odds ratios with 95% confidence intervals and two-sided *p*-values. Model discrimination was assessed using the area under the receiver operating characteristic curve with a 95% confidence interval. Calibration was evaluated using the Hosmer–Lemeshow goodness-of-fit test and the Brier score. The final model demonstrated an AUC of 0.736 (95% CI, 0.685–0.787), a Hosmer–Lemeshow *p*-value of 0.219, and a Brier score of 0.208.

Potential multicollinearity was assessed using variance inflation factors, tolerance values, and the condition index. Values suggesting potentially important collinearity were defined a priori as a variance inflation factor greater than 5, tolerance below 0.20, or a condition index greater than 15. The maximum variance inflation factor was 1.49, the minimum tolerance was 0.67, and the maximum condition index was 2.34.

The regression coefficients were interpreted as mutually adjusted associations with a physician-guided treatment decision. Variables such as LDL-C concentration and history of myocardial infarction are established elements of clinical decision-making regarding lipid-lowering therapy. Consequently, the model was intended to characterize real-world prescribing patterns and the extent to which recognized clinical characteristics were reflected in treatment initiation. It was not intended to identify novel biological or mechanistic determinants, establish causal relationships, or evaluate the clinical effectiveness of statin therapy.

All the statistical tests were two-tailed, and a *p*-value < 0.05 was considered statistically significant.

## 3. Results

A total of 374 previously statin-naïve patients hospitalized for decompensated heart failure were included in the analysis. Statin therapy was not initiated during the index hospitalization in 206 patients, whereas treatment was initiated in 168 patients. Table 1 presents the detailed comparison of demographic, clinical, laboratory, and therapeutic characteristics between the two study groups.

Patients in whom statin therapy was initiated were younger than those in whom statin therapy was not initiated (65.1 [52.3–73.4] vs. 69.8 [60.2–77.5] years; *p* = 0.028). No significant between-group differences were observed in sex distribution (77.4% vs. 70.4% male; *p* = 0.127), alcohol consumption (*p* = 0.395), smoking status (*p* = 0.122), or BMI (*p* = 0.155).

Arterial hypertension was more prevalent in the statin-initiation group than in the no-statin-initiation group (62.5% vs. 50.5%; *p* = 0.026). A history of myocardial infarction was also more frequent among patients in whom statin therapy was initiated (20.8% vs. 12.6%; *p* = 0.033). The distribution of atrial fibrillation subtypes did not differ between the groups (*p* = 0.878). Paroxysmal atrial fibrillation was present in 14.9% of patients in the statin-initiation group and 15.5% of those in the no-statin-initiation group, whereas persistent or permanent atrial fibrillation was present in 33.9% and 35.9%, respectively. No significant differences were observed in the prevalence of stable angina, previous percutaneous coronary intervention, previous coronary artery bypass grafting, stroke, transient ischemic attack, diabetes mellitus, chronic kidney disease, anemia, iron deficiency, thyroid dysfunction, malignancy, or depression.

The overall distribution of heart failure etiologies did not differ significantly between the groups (*p* = 0.086), although ischemic heart disease was numerically more frequent in the statin-initiation group than in the no-statin-initiation group (32.1% vs. 21.8%).

After cardiovascular risk categories were reassigned using a hierarchical and mutually exclusive approach, 79 patients (47.0%) in the statin-initiation group and 71 patients (34.5%) in the no-statin-initiation group were classified as being at very high cardiovascular risk. High cardiovascular risk was present in 38 patients (22.6%) and 33 patients (16.0%), respectively, whereas low or moderate cardiovascular risk was identified in 51 patients (30.4%) and 102 patients (49.5%), respectively. The overall distribution of cardiovascular risk categories differed significantly between the groups (*p* < 0.001).

Established ASCVD, corresponding to a registry-identifiable secondary-prevention indication, was present in 79 patients (47.0%) in the statin-initiation group and 71 patients (34.5%) in the no-statin-initiation group. Among patients without established ASCVD, a registry-identifiable primary-prevention indication was identified in 60 patients (35.7%) and 63 patients (30.6%), respectively. No apparent guideline-based indication for statin therapy could be identified from the available registry data in 29 patients (17.3%) in the statin-initiation group and 72 patients (35.0%) in the no-statin-initiation group. The overall distribution of these hierarchical and mutually exclusive indication categories differed significantly between the groups (*p* < 0.001).

Baseline LDL-C and total cholesterol concentrations were significantly higher among patients in whom statin therapy was initiated than among those in whom it was not initiated (LDL-C: 95.0 [72.0–127.75] vs. 80.5 [58.75–104.25] mg/dL; *p* < 0.001; total cholesterol: 185 [155–210] vs. 155 [128–180] mg/dL; *p* < 0.001). No significant between-group differences were observed in HDL-C, triglycerides, liver enzymes, glycemic parameters, blood cell counts, inflammatory markers, serum creatinine, NT-proBNP, troponin I, electrolytes, thyroid-stimulating hormone, or uric acid.

Systolic and diastolic blood pressure at admission were higher in the statin-initiation group (systolic blood pressure: 133.5 [120.0–148.25] vs. 125.0 [110.0–140.0] mmHg; *p* < 0.001; diastolic blood pressure: 78 [70–86] vs. 74 [66–82] mmHg; *p* = 0.009), whereas heart rate was slightly lower (78 [68–88] vs. 82 [72–94] beats/min; *p* = 0.048). Patients in whom statin therapy was initiated also had shorter Timed Up and Go test times (10.2 [8.5–15.0] vs. 12.8 [9.5–20.4] s; *p* = 0.039).

The KCCQ-12 summary score did not differ significantly between the groups, although it was numerically lower in the statin-initiation group (39.6 [26.3–59.8] vs. 48.7 [23.9–74.1]; *p* = 0.108). No significant difference was observed in the visual analogue quality-of-life score (*p* = 0.238).

The distribution of Canadian Cardiovascular Society angina classes differed significantly between the groups (*p* < 0.001). CCS class I was more frequent in the no-statin-initiation group than in the statin-initiation group (86.0% vs. 44.0%), whereas CCS classes II–IV were more frequently recorded in the statin-initiation group. In contrast, the distribution of New York Heart Association functional classes did not differ significantly between the groups (*p* = 0.754).

Median LVEF did not differ significantly between patients in whom statin therapy was initiated and those in whom it was not initiated (31.5 [28.0–42.0]% vs. 33.0 [24.25–50.0]%; *p* = 0.548). HFpEF was present in 21 patients (12.5%) in the statin-initiation group and 42 patients (20.4%) in the no-statin-initiation group. HFmrEF was present in 56 patients (33.3%) and 61 patients (29.6%), respectively, whereas HFrEF was present in 91 patients (54.2%) and 103 patients (50.0%), respectively. The overall distribution of heart failure phenotypes according to LVEF did not differ significantly between the groups (*p* = 0.126).

Regarding baseline pharmacological treatment, ARNI therapy was more frequent in the statin-initiation group than in the no-statin-initiation group (36.9% vs. 22.3%; *p* = 0.002), whereas ARB therapy was less frequent (7.1% vs. 15.0%; *p* = 0.017). SGLT2 inhibitor use was also less frequent in the statin-initiation group (15.5% vs. 27.7%; *p* = 0.005). Anticoagulant therapy was more frequent among patients in whom statin therapy was initiated (44.6% vs. 29.6%; *p* = 0.003). MRA therapy was numerically less frequent in the statin-initiation group, with a borderline between-group difference (29.2% vs. 38.8%; *p* = 0.050). No significant differences were observed in the use of ACE inhibitors, beta-blockers, diuretics, antiplatelet therapy, or nitrates. The median GDMT score did not differ significantly between the groups (1.00 [0.00–2.25] vs. 2.00 [0.25–3.00]; *p* = 0.108).

No statistically significant between-group differences were observed in treatments administered during hospitalization, including diuretics, inotropic or vasoactive agents, mechanical circulatory support, and noninvasive or invasive mechanical ventilation. These comparisons were descriptive and should not be interpreted as demonstrating that concomitant therapies causally determined the decision to initiate statin treatment.

Table 2 presents the results of the prespecified multivariable logistic regression model evaluating factors associated with in-hospital statin initiation among previously statin-naïve patients hospitalized for acute decompensated heart failure. All the variables included in the model are reported irrespective of their statistical significance.

After multivariable adjustment, each 1-year increase in age was associated with a 3% reduction in the odds of statin initiation (aOR, 0.97; 95% CI, 0.95–0.99; *p* = 0.008). Male sex (aOR, 2.01; 95% CI, 1.12–3.61; *p* = 0.019), arterial hypertension (aOR, 1.65; 95% CI, 1.06–2.56; *p* = 0.023), and a history of myocardial infarction (aOR, 1.82; 95% CI, 1.03–3.22; *p* = 0.040) were associated with higher odds of treatment initiation.

Each 10 mmHg increase in systolic blood pressure was associated with a 12% increase in the adjusted odds of statin initiation (aOR, 1.12; 95% CI, 1.01–1.25; *p* = 0.034), whereas each 10 mg/dL increase in LDL-C was associated with a 15% increase (aOR, 1.15; 95% CI, 1.07–1.22; *p* < 0.001).

Each 10-point increase in the KCCQ-12 score was associated with a 12% reduction in the adjusted odds of statin initiation (aOR, 0.88; 95% CI, 0.81–0.96; *p* = 0.005). Anticoagulant therapy was associated with higher adjusted odds of statin initiation (aOR, 1.88; 95% CI, 1.20–2.96; *p* = 0.006).

**Figure 2:**
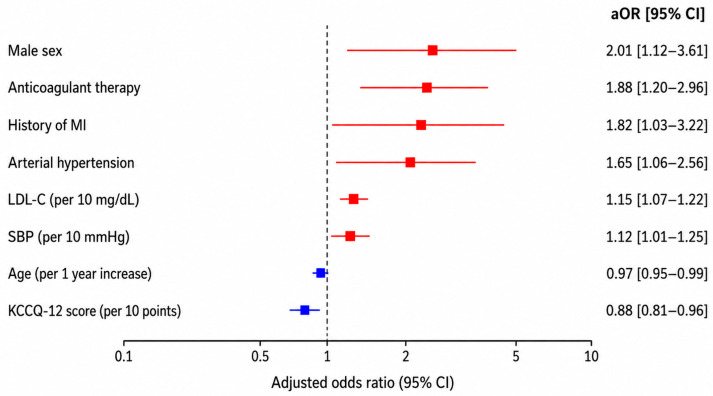
Forest plot of statistically significant factors associated with in-hospital statin initiation in the prespecified multivariable logistic regression model. Squares indicate adjusted odds ratios, and horizontal lines indicate 95% confidence intervals. The vertical dashed line denotes aOR = 1.0. Abbreviations: aOR, adjusted odds ratio; CI, confidence interval; KCCQ-12, 12-item Kansas City Cardiomyopathy Questionnaire; LDL-C, low-density lipoprotein cholesterol; MI, myocardial infarction; SBP, systolic blood pressure.

LVEF (aOR per 5-percentage-point increase, 0.93; 95% CI, 0.85–1.02; *p* = 0.143) and the GDMT score (aOR per additional component, 0.83; 95% CI, 0.64–1.09; *p* = 0.184) were not significantly associated with treatment initiation. The statistically significant associations are summarized graphically in Figure 2.

## 4. Discussion

This multicenter study characterized real-world patterns of in-hospital statin initiation among 374 previously statin-naïve patients hospitalized for acute decompensated heart failure. After multivariable adjustment, younger age, male sex, arterial hypertension, a history of myocardial infarction, higher systolic blood pressure, higher LDL-C concentration, a lower KCCQ-12 score, and anticoagulant therapy were associated with a higher odds of statin initiation. In contrast, LVEF and the GDMT score were not significantly associated with this treatment decision. These findings should be interpreted exclusively as mutually adjusted correlates of physician-guided prescribing behavior. They do not establish causal or mechanistic determinants of statin initiation, demonstrate treatment benefit, or provide a basis for recommending statin therapy solely because of ADHF.

The associations with LDL-C concentration and previous myocardial infarction are clinically expected because both variables are established components of cardiovascular risk assessment and routine decision-making regarding lipid-lowering therapy [3,9]. Their presence in the model therefore reflects the extent to which recognized preventive considerations were incorporated into clinical practice rather than the identification of novel biological relationships. To reduce conceptual circularity and redundancy, cardiovascular risk category was excluded from the revised regression model because it was partly derived from clinical characteristics already represented by individual covariates. Similarly, LDL-C was retained as the principal lipid variable without simultaneous adjustment for total cholesterol.

The descriptive analysis showed that patients in whom statin therapy was initiated more frequently belonged to very-high or high cardiovascular risk categories and more commonly had a registry-identifiable secondary- or primary-prevention indication. Nevertheless, established ASCVD was present in 34.5% of patients in whom statin therapy was not initiated, while a further 30.6% had characteristics potentially supporting statin therapy for primary prevention. Hospitalization for worsening heart failure provides an opportunity to reassess cardiovascular comorbidities, modifiable risk factors, and preventive pharmacotherapy [2,3,9,13]. However, this apparent discordance should not automatically be interpreted as inappropriate care, therapeutic inertia or evidence that statin therapy should have been initiated in individual patients. The registry did not systematically record previous statin intolerance, contraindications, drug interactions, frailty, limited life expectancy, patient preferences, or plans to initiate lipid-lowering therapy after discharge. The findings may identify areas in which a structured reassessment of preventive indications could be considered, but they cannot establish the appropriateness of individual prescribing decisions. Individualized treatment selection in heart failure requires consideration of comorbidities, functional status, prognosis, treatment tolerance, and competing therapeutic priorities [15].

The present findings should be considered in the context of previous evidence. The CORONA and GISSI-HF trials did not demonstrate a significant reduction in mortality or major cardiovascular outcomes when rosuvastatin was used specifically as treatment for chronic systolic heart failure [7,8]; accordingly, current European and American guidelines do not recommend routine statin initiation solely because of heart failure in the absence of another established indication [3,9]. Observational evidence has suggested possible benefits in selected populations, including patients with LVEF ≥ 50% [16], but such associations remain susceptible to confounding by indication and treatment-selection bias. Consequently, randomized evidence is still needed before statin initiation can be recommended specifically as a therapeutic strategy for HFpEF [17].

Lipid concentrations and statin exposure in heart failure require cautious interpretation. A systematic review and meta-analysis indicated that lipid parameters may carry prognostic information in patients with heart failure [18], while earlier data associated low total cholesterol with increased mortality in advanced disease, potentially reflecting inflammation, malnutrition, cardiac cachexia, or greater disease severity [19]. More recent observational analyses have reported associations between statin therapy and improved long-term outcomes after hospitalization for acute heart failure [20], including lower all-cause mortality in HFpEF [21] and potentially different associations with mortality and rehospitalization across LVEF phenotypes [22]. These studies evaluated prognosis associated with statin exposure, whereas the present analysis examined characteristics associated with a decision to initiate treatment and cannot determine the clinical effectiveness of that decision.

In the present cohort, neither median LVEF nor the distribution of HFpEF, HFmrEF, and HFrEF differed significantly between the study groups, and LVEF was not independently associated with statin initiation. These findings do not support an independent relationship between heart failure phenotype or the degree of left ventricular systolic dysfunction and the in-hospital prescribing decision. Older age was associated with lower adjusted odds of statin initiation, but age itself should not be interpreted as a contraindication to lipid-lowering therapy. This association may instead reflect unmeasured frailty, multimorbidity, prognosis, medication burden, competing treatment priorities, or patient preference. The inverse association with KCCQ-12 should likewise be regarded as exploratory because the score did not differ significantly in the unadjusted comparison and is not a conventional determinant of statin use. Similarly, anticoagulant therapy may represent a marker of cardiovascular comorbidity or broader prescribing patterns rather than a direct determinant of statin initiation. The absence of an association with the GDMT score does not support the interpretation that statin initiation was simply part of a generalized pattern of more intensive heart failure pharmacotherapy.

The principal strength of this study is the analysis of a multicenter cohort restricted to patients who were statin-naïve at admission. This approach reduced confounding related to the continuation of pre-existing lipid-lowering therapy and enabled a focused assessment of factors associated with a new in-hospital prescribing decision. Detailed demographic, clinical, laboratory, echocardiographic, functional, and pharmacological data also allowed statin initiation to be evaluated within a broad cardiovascular context.

Several limitations should be acknowledged. The observational design precludes causal interpretation, and residual confounding cannot be excluded. The registry did not systematically record the reasons for initiating or withholding statin therapy, including intolerance, contraindications, clinically relevant interactions, frailty, life expectancy, patient preference, or planned post-discharge treatment. Prescribing decisions may also have been influenced by individual physician preferences, institutional practice patterns, and local healthcare policies, none of which could be adequately captured in this registry-based analysis. Information on the specific statin, treatment intensity, dose, adverse effects, persistence, and adherence was also unavailable. Consequently, the analysis reflects the in-hospital prescribing decision rather than confirmed long-term exposure to lipid-lowering therapy. Complete and uniformly collected follow-up data on all-cause mortality and heart failure rehospitalization were unavailable for the entire cohort; therefore, reliable Kaplan–Meier analyses, Cox proportional hazards models, and outcome-based subgroup analyses could not be performed without a substantial risk of selection bias and unstable estimates. The findings originate from a single national healthcare system and may not be fully generalizable. Finally, although the multivariable model demonstrated acceptable discrimination and reassuring collinearity diagnostics, it was developed to characterize treatment patterns rather than to serve as a validated clinical prediction tool.

## 5. Conclusions

Among previously statin-naïve patients hospitalized for acute decompensated heart failure, in-hospital statin initiation was associated with a combination of demographic, clinical, lipid-related, hemodynamic, functional, and treatment-related characteristics. These variables should be interpreted as correlates of real-world physician-guided prescribing decisions rather than as causal determinants of statin initiation or predictors of clinical benefit.

The findings suggest that established preventive considerations, including LDL-C concentration and a history of myocardial infarction, were reflected in treatment decisions. However, a substantial proportion of patients with registry-identifiable secondary- or primary-prevention indications did not have statin therapy initiated during the index hospitalization. Because the reasons for initiating or withholding treatment and complete longitudinal outcome data were unavailable, the appropriateness and clinical consequences of individual prescribing decisions could not be determined.

Prospective studies with systematically documented treatment rationale and complete follow-up are needed to evaluate the clinical implications of these prescribing patterns.

## Figures and Tables

**Table 1:** Clinical, laboratory, echocardiographic, and treatment characteristics of patients according to in-hospital statin initiation.

Variable		Study Groups		*p*-Value
		No Statins (*n* = 206)	Statin Initiation (*n* = 168)	
Sex	male, *n* (%)	145 (70.4%)	130 (77.4%)	0.127
	female, *n* (%)	61 (29.6%)	38 (22.6%)	
Age, years	Median [Q1–Q3]	69.8 [60.2–77.5]	65.1 [52.3–73.4]	0.028
alcohol consumption	never, *n* (%)	165 (80.1%)	134 (79.8%)	0.395
	current, *n* (%)	13 (6.3%)	16 (9.5%)	
	former, *n* (%)	28 (13.6%)	18 (10.7%)	
cigarette smoking	never, *n* (%)	102 (49.5%)	66 (39.3%)	0.122
	current, *n* (%)	37 (18.0%)	40 (23.8%)	
	former, *n* (%)	67 (32.5%)	62 (36.9%)	
BMI, kg/m^2^	Median [Q1–Q3]	26.92 [23.4–31.47]	27.76 [24.62–32.28]	0.155
Underweight: BMI < 18.5	*n* (%)	9 (4.4%)	3 (1.8%)	
Normal weight: BMI 18.5–24.9	*n* (%)	65 (31.6%)	45 (26.8%)	
Overweight: BMI 25.0–29.9	*n* (%)	66 (32.0%)	65 (38.7%)	
Obesity: BMI ≥ 30.0	*n* (%)	66 (32.0%)	55 (32.7%)	
Comorbidities				
Arterial hypertension	*n* (%)	104 (50.5%)	105 (62.5%)	0.026
Atrial fibrillation	paroxysmal, *n* (%)	32 (15.5%)	25 (14.9%)	0.878
	persistent/permanent, *n* (%)	74 (35.9%)	57 (33.9%)	
Myocardial infarction	*n* (%)	26 (12.6%)	35 (20.8%)	0.033
Stable angina	*n* (%)	30 (14.6%)	29 (17.3%)	0.476
PCI	*n* (%)	26 (12.6%)	24 (14.3%)	0.638
CABG	*n* (%)	9 (4.4%)	11 (6.5%)	0.352
Carotid artery disease	*n* (%)	0 (0%)	1 (0,6%)	0.449
LAED	*n* (%)	4 (1.9%)	9 (5.4%)	0.073
Limb amputation	*n* (%)	0 (0%)	1 (0.6%)	0.449
Thyroid	hyperthyroidism, *n* (%)	22 (10.7%)	10 (5.9%)	0.254
	hypothyroidism, *n* (%)	8 (3.9%)	8 (4.8%)	
Diabetes	diet, *n* (%)	8 (3.9%)	6 (3.6%)	0.218
	insulin, *n* (%)	16 (7.8%)	8 (4.8%)	
	oral drugs, *n* (%)	31 (15.1%)	38 (22.6%)	
Chronic kidney disease	*n* (%)	37 (17.9%)	40 (23.8%)	0.164
Dialysis	*n* (%)	6 (2.9%)	2 (1.2%)	0.304
Anemia	*n* (%)	28 (13.6%)	17 (10.1%)	0.304
Iron deficiency	*n* (%)	19 (9.2%)	16 (9.5%)	0.921
Stroke	*n* (%)	13 (6.3%)	14 (8.3%)	0.452
TIA	*n* (%)	3 (1.5%)	1 (0.6%)	0.631
Cancer	*n* (%)	14 (6.8%)	14 (8.3%)	0.574
Depression	*n* (%)	10 (4.9%)	3 (1.8%)	0.107
Laboratory tests at baseline				
LDL-C, mg/dL	Median [Q1–Q3]	80.50 [58.75–104.25]	95.00 [72.00–127.75]	<0.001
Total cholesterol, mg/dL	Median [Q1–Q3]	155 [128–180]	185 [155–210]	<0.001
TG, mg/dL,	Median [Q1–Q3]	92.5 [71.75–130]	99.88 [69.75–139.25]	0.331
HDL-C, mg/dL	Median [Q1–Q3]	43 [33–53]	44 [34–52]	0.788
ALT, U/L	Median [Q1–Q3]	26 [19–42]	28 [18–45]	0.872
AST, U/L	Median [Q1–Q3]	30 [22–44.1]	33 [24–47.5]	0.152
Bilirubin, mg/dL	Median [Q1–Q3]	1 [0.63–1.57]	0.97 [0.61–1.74]	0.599
HbA1c, %	Median [Q1–Q3]	5.89 [5.43–6.48]	5.9 [5.5–6.61]	0.518
Fasting glucose, mg/dL	Median [Q1–Q3]	101 [90.25–126]	107 [92.5–151.5]	0.116
Hemoglobin, g/dL	Median [Q1–Q3]	13.71 [11.95–14.95]	14.1 [12.63–15.1]	0.185
PLT, ×10^3^/µL	Median [Q1–Q3]	210 [170–260]	220 [185–275]	0.270
WBC, ×10^3^/µL	Median [Q1–Q3]	8.4 [4.2–17.8]	9.5 [5.0–18.9]	0.420
CRP, mg/L	Median [Q1–Q3]	8.1 [6.9–10.4]	7.9 [6.5–9.8]	0.310
Procalcitonin, ng/mL	Median [Q1–Q3]	0.13 [0.04–0.33]	0,07 [0.02–0.34]	0.321
NT-proBNP, pg/mL	Median [Q1–Q3]	3752.6 [902–8237.5]	3702 [1666.5–7775]	0.536
Troponin I, ng/mL	Median [Q1–Q3]	1.9 [1.38–26.9]	3.2 [0.02–40.25]	0.271
Creatinine, mg/dL	Median [Q1–Q3]	1.16 [0.91–1.48]	1.16 [0.92–1.67]	0.263
Sodium, mmol/L	Median [Q1–Q3]	139 [137–142]	139.8 [137.7–141.2]	0.825
Potassium, mmol/L	Median [Q1–Q3]	4.4 [4.13–4.81]	4.4 [4–4.78]	0.369
TSH, µIU/mL	Median [Q1–Q3]	1.73 [0.82–2.63]	1.7 [0.87–2.26]	0.755
Uric acid, mg/dL	Median [Q1–Q3]	7.45 [5.38–10.4]	7.55 [5.98–9.83]	0.536
Heart failure etiology				
ischemic heart disease	*n* (%)	45 (21.8%)	54 (32.1%)	0.190
cardiomyopathy	*n* (%)	33 (16.0%)	27 (16.1%)	
arterial hypertension	*n* (%)	32 (15.5%)	25 (14.9%)	
arrhythmia cardiomyopathy	*n* (%)	26 (12.6%)	10 (6.0%)	
suspected toxic cardiomyopathy	*n* (%)	11 (5.3%)	6 (3.6%)	
significant valve disease	*n* (%)	24 (11.7%)	18 (10.7%)	
other	*n* (%)	35 (17.0%)	28 (16.0%)	
Symptoms				
Blood pressure systolic, mm Hg	Median [Q1–Q3]	125.00 [110.00–140.00]	133.50 [120.00–148.25]	<0.001
Blood pressure diastolic, mm Hg	Median [Q1–Q3]	74 [66–82]	78 [70–86]	0.009
Heart rate, beats/min	Median [Q1–Q3]	82 [72–94]	78 [68–88]	0.048
Weight gain at hospitalization	*n* (%)	33 (16.0%)	22 (13.1%)	0.427
Activity TUG test	Median [Q1–Q3]	12.8 [9.5–20.4]	10.2 [8.5–15.0]	0.039
CCS class				
I	*n* (%)	177 (86.0%)	74 (44.0%)	<0.001
II	*n* (%)	17 (8.2%)	71 (42.3%)	
III	*n* (%)	8 (3.8%)	11 (6.5%)	
IV	*n* (%)	4 (2.0%)	12 (7.2%)	
NYHA class				
I	*n* (%)	66 (32.0%)	46 (27.4%)	0.754
II	*n* (%)	117 (56.8%)	100 (59.5%)	
III	*n* (%)	19 (9.2%)	19 (11.3%)	
IV	*n* (%)	4 (2.0%)	3 (1.8)	
CV risk				
Very high	*n* (%)	71 (34.5%)	79 (47.0%)	<0.001
High	*n* (%)	33 (16.00%)	38 (22.6%)	
Low or moderate	*n* (%)	102 (49.5%)	51 (30.4%)	
Apparent indication category				
Established ASCVD—secondary prevention	*n* (%)	71 (34.5%)	79 (47.0%)	<0.001
Primary-prevention indication without established ASCVD	*n* (%)	63 (30.6%)	60 (35.7%)	
No apparent indication identifiable from registry data	*n* (%)	72 (35.0%)	29 (17.3%)	
KCCQ12 Summary Score (KCCQ12-SS)	Median [Q1–Q3]	48.7 [23.9–74.1]	39.6 [26.3–59.8]	0.108
Visual analogue quality of life score	Median [Q1–Q3]	50 [40–70]	50 [40–75]	0.238
Echocardiographic findings				
LVEF, %	Median [Q1–Q3]	33.00 [24.2–50.0]	31.50 [28.0–42.0]	0.548
HFpEF, LVEF ≥ 50%	*n* (%)	42 (20.4%)	21 (12.5%)	0.126
HFmrEF, LVEF 41–49%	*n* (%)	61 (29.6%)	56 (33.3%)	
HFrEF, LVEF ≤ 40%	*n* (%)	103 (50.0%)	91 (54.2%)	
Medical treatment				
ACEI	*n* (%)	63 (30.6%)	51 (30.4%)	0.962
ARB	*n* (%)	31 (15.0%)	12 (7.1%)	0.017
ARNI	*n* (%)	46 (22.3%)	62 (36.9%)	0.002
MRA	*n* (%)	80 (38.8%)	49 (29.2%)	0.050
Beta-blocker	*n* (%)	134 (65.0%)	96 (57.1%)	0.118
Diuretics	*n* (%)	104 (50.5%)	79 (47.0%)	0.505
SGLT2 inhibitors	*n* (%)	57 (27.7%)	26 (15.5%)	0.005
Antiplatelet therapy	*n* (%)	29 (14.1%)	28 (16.7%)	0.488
Anticoagulant therapy	*n* (%)	61 (29.6%)	75 (44.6%)	0.003
Nitrates	*n* (%)	1 (0.5%)	2 (1.2%)	0.603
GDMT score	Median [Q1–Q3]	2.00 [0.25–3.00]	1.00 [0.00–2.25]	0.108
Treatment during hospitalization				
Diuretics	*n* (%)	106 (51.5%)	103 (61.3%)	0.056
Dobutamine	*n* (%)	17 (8.3%)	21 (12.5%)	0.176
Dopamine	*n* (%)	7 (3.4%)	2 (1.2%)	0.195
Epinephrine	*n* (%)	5 (2.4%)	1 (0.6%)	0.230
Levosimendan	*n* (%)	2 (1.0%)	1 (0.6%)	1.000
Milrinone	*n* (%)	0 (0%)	1 (0.6%)	0.449
Norepinephrine	*n* (%)	6 (2.9%)	8 (4.8%)	0.349
IABP	*n* (%)	1 (0.5%)	3 (1.8%)	0.330
LVAD	*n* (%)	2 (1.0%)	2 (1.2%)	1.000
Noninvasive Mechanical Ventilation	*n* (%)	1 (0.5%)	2 (1.2%)	0.590
Invasive Mechanical Ventilation	*n* (%)	8 (3.9%)	9 (5.4%)	0.496

ACEI, angiotensin-converting enzyme inhibitor; AF, atrial fibrillation; ALT, alanine aminotransferase; ARB, angiotensin II receptor blocker; ARNI, angiotensin receptor–neprilysin inhibitor; AST, aspartate aminotransferase; BMI, body mass index; CABG, coronary artery bypass grafting; CCS, Canadian Cardiovascular Society; CRP, C-reactive protein; CV, cardiovascular; ECHO, echocardiography; GDMT, guideline-directed medical therapy; HbA1c, glycated hemoglobin; HDL, high-density lipoprotein cholesterol; HF, heart failure; HFmrEF, heart failure with mildly reduced ejection fraction; HFpEF, heart failure with preserved ejection fraction; HFrEF, heart failure with reduced ejection fraction; IABP, intra-aortic balloon pump; KCCQ12-SS, 12-item Kansas City Cardiomyopathy Questionnaire Summary Score; LAED, lower extremity artery disease; LDL-C, low-density lipoprotein cholesterol; LVAD, left ventricular assist device; LVEF, left ventricular ejection fraction; MRA, mineralocorticoid receptor antagonist; NT-proBNP, N-terminal pro-B-type natriuretic peptide; NYHA, New York Heart Association; PCI, percutaneous coronary intervention; PLT, platelet count; SGLT2i, sodium–glucose cotransporter 2 inhibitor; TIA, transient ischemic attack; TG, triglycerides; TSH, thyroid-stimulating hormone; TUG, Timed Up and Go; WBC, white blood cell count.

**Table 2:** Prespecified multivariable logistic regression model of factors associated with in-hospital statin initiation among previously statin-naïve patients.

Variable	Adjusted Odds Ratio (aOR)	95% Confidence Interval	*p*-Value
Age (per 1 year)	0.97	0.95–0.99	0.008
Male sex	2.01	1.12–3.61	0.019
Arterial hypertension	1.65	1.06–2.56	0.023
History of myocardial infarction	1.82	1.03–3.22	0.040
Systolic blood pressure, per 10 mmHg increase	1.12	1.01–1.25	0.034
LDL-C, per 10 mg/dL increase	1.15	1.07–1.22	<0.001
LVEF, per 5-percentage-point increase	0.93	0.85–1.02	0.143
KCCQ-12 score, per 10-point increase	0.88	0.81–0.96	0.005
GDMT score, per additional component	0.83	0.64–1.09	0.184
Anticoagulant therapy	1.88	1.20–2.96	0.006

aOR, adjusted odds ratio; CI, confidence interval; GDMT, guideline-directed medical therapy; KCCQ-12, 12-item Kansas City Cardiomyopathy Questionnaire; LDL-C, low-density lipoprotein cholesterol; LVEF, left ventricular ejection fraction; SBP, systolic blood pressure.

## Data Availability

The data presented in this study are available upon request from the first author. The data are not publicly available due to ethical restrictions. The availability of the data underpinning this study can be accessed upon request from the corresponding author.

## Abbreviations

ADHF	acute decompensated heart failure
aOR	adjusted odds ratio
ARNI	angiotensin receptor–neprilysin inhibitor
BP	blood pressure
CV	cardiovascular
HEROES	HEart failuRe ObsErvational Study
LDL-C	low-density lipoprotein cholesterol
MRA	mineralocorticoid receptor antagonist
MI	myocardial infarction
SBP	systolic blood pressure
SGLT2i	sodium–glucose cotransporter 2 inhibitor

## References

[1] Shahim B., Kapelios C.J., Savarese G., Lund L.H. (2023). Global Public Health Burden of Heart Failure: An Updated Review. Card. Fail. Rev..

[2] Greene S.J., Bauersachs J., Brugts J.J., Ezekowitz J.A., Lam C.S.P., Lund L.H., Ponikowski P., Voors A.A., Zannad F., Zieroth S. (2023). Worsening Heart Failure: Nomenclature, Epidemiology, and Future Directions: JACC Review Topic of the Week. J. Am. Coll. Cardiol..

[3] McDonagh T.A., Metra M., Adamo M., Gardner R.S., Baumbach A., Böhm M., Burri H., Butler J., Čelutkienė J., Chioncel O. (2023). 2023 Focused Update of the 2021 ESC Guidelines for the diagnosis and treatment of acute and chronic heart failure. Eur. Heart J..

[4] Choudhary A., Rawat U., Kumar P., Mittal P. (2023). Pleotropic effects of statins: The dilemma of wider utilization of statin. Egypt. Heart J..

[5] Correale M., Abruzzese S., Greco C.A., Concilio M., Biase M.D., Brunetti N.D. (2014). Pleiotropic effects of statin in therapy in heart failure: A review. Curr. Vasc. Pharmacol..

[6] Orkaby A.R., Goyal P., Charest B., Qazi S., Sheikh S., Shah S., Gaziano J.M., Djousse L., Gagnon D., Joseph J. (2024). Initiation of Statins for Primary Prevention in Heart Failure with Preserved Ejection Fraction. JACC Adv..

[7] Kjekshus J., Apetrei E., Barrios V., Böhm M., Cleland J.G., Cornel J.H., Dunselman P., Fonseca C., Goudev A., Grande P. (2007). Rosuvastatin in older patients with systolic heart failure. N. Engl. J. Med..

[8] Tavazzi L., Maggioni A.P., Marchioli R., Barlera S., Franzosi M.G., Latini R., Lucci D., Nicolosi G.L., Porcu M., Tognoni G. (2008). Effect of rosuvastatin in patients with chronic heart failure (the GISSI-HF trial): A randomised, double-blind, placebo-controlled trial. Lancet.

[9] Heidenreich P.A., Bozkurt B., Aguilar D., Allen L.A., Byun J.J., Colvin M.M., Deswal A., Drazner M.H., Dunlay S.M., Evers L.R. (2022). 2022 AHA/ACC/HFSA Guideline for the Management of Heart Failure: A Report of the American College of Cardiology/American Heart Association Joint Committee on Clinical Practice Guidelines. J. Am. Coll. Cardiol..

[10] Zheng X., Tan L., Zhang Y. (2024). The impact of statin use on short-term and long-term mortality in patients with heart failure. Front. Pharmacol..

[11] Wang L., Wang Y. (2025). Statin therapy and mortality in critically ill heart failure patients: Insights from a triangulated real-world design study. PLoS ONE.

[12] Nochioka K., Sakata Y., Miyata S., Miura M., Takada T., Tadaki S., Ushigome R., Yamauchi T., Takahashi J., Shimokawa H. (2015). CHART-2 Investigators. Prognostic impact of statin use in patients with heart failure and preserved ejection fraction. Circ. J..

[13] Mach F., Koskinas K.C., Roeters van Lennep J.E., Tokgözoğlu L., Badimon L., Baigent C., Benn M., Binder C.J., Catapano A.L., De Backer G.G. (2025). Focused update 2025 delle linee guida ESC/EAS 2019 per il trattamento delle dislipidemie [2025 Focused Update of the 2019 ESC/EAS Guidelines for the management of dyslipidaemias]. G. Ital. Cardiol..

[14] Drożdż J., Morawiec R., Drozd M., Krzesiński P., Wożakowska-Kapłon B., Grabowski M., Leszek P., Kuch M., Kasprzak J.D., Janion M. (2025). Rationale, objectives, and design of the HEart failuRe ObsErvational Study of the Polish Cardiac Society (HEROES). Kardiol. Pol..

[15] Rosano G.M.C., Moura B., Metra M., Böhm M., Bauersachs J., Ben Gal T., Adamopoulos S., Abdelhamid M., Bistola V., Čelutkienė J. (2021). Patient profiling in heart failure for tailoring medical therapy. A consensus document of the Heart Failure Association of the European Society of Cardiology. Eur. J. Heart Fail..

[16] Alehagen U., Benson L., Edner M., Dahlström U., Lund L.H. (2015). Association Between Use of Statins and Mortality in Patients with Heart Failure and Ejection Fraction of ≥50. Circ. Heart Fail..

[17] Sundaram V., Karnib M., Selvaganesan P. (2024). Statin Therapy in Heart Failure with Preserved Ejection Fraction: The Need for Randomized Evidence. JACC Adv..

[18] Sayed A., Afify H., Munir M., ElGarhy I., Shazly O., ElRefaei M., Ahmed S., Amin A.M., Amine O.C., Elgendy I.Y. (2025). Prognostic value of lipid parameters among patients with heart failure: A systematic review and meta-analysis. ESC Heart Fail..

[19] Horwich T.B., Hamilton M.A., Maclellan W.R., Fonarow G.C. (2002). Low serum total cholesterol is associated with marked increase in mortality in advanced heart failure. J. Card. Fail..

[20] Monayer A., Minha S., Maymon S.L., Pereg D., Kalmanovich E., Moravsky G., Grupper A., Marcus G. (2024). Statin therapy impact on Long-Term outcomes in acute heart Failure: Retrospective analysis of hospitalized patients. Int. J. Cardiol. Heart Vasc..

[21] Kaur G., Jones M., Howes L., Hattingh H.L. (2023). Systematic review and meta-analysis of the association between all-cause mortality and statin therapy in patients with preserved ejection fraction heart failure (HFpEF). Int. J. Cardiol..

[22] Al-Jarallah M., Rajan R., Dashti R., Bulbanat B., Ridha M., Sulaiman K., Al-Zakwani I., Alsheikh-Ali A.A., Panduranga P., Alhabib K.F. (2026). Impact of Statin Therapy on Mortality and Rehospitalization in Acute Heart Failure Patients Stratified by Ejection Fraction: Insights from the Gulf CARE Registry. Curr. Vasc. Pharmacol..

